# Willingness to volunteer and readiness to practice of undergraduate medical students during the COVID-19 pandemic: a cross-sectional survey in Indonesia

**DOI:** 10.1186/s12909-021-02576-0

**Published:** 2021-03-01

**Authors:** Gilbert Lazarus, Ardi Findyartini, Azis Muhammad Putera, Nico Gamalliel, David Nugraha, Imam Adli, Jason Phowira, Lyanna Azzahra, Bagas Ariffandi, Indah Suci Widyahening

**Affiliations:** 1 Faculty of Medicine, Universitas Indonesia, Jakarta, Indonesia; 2 Department of Medical Education, Faculty of Medicine, Universitas Indonesia, Salemba 6, Central, Jakarta, 10430 Indonesia; 3 Medical Education Center, Indonesia Medical Education and Research Institute, Faculty of Medicine, Universitas Indonesia, Jakarta, Indonesia; 4 Faculty of Medicine, Universitas Airlangga, Surabaya, Indonesia; 5 Department of Community Medicine, Faculty of Medicine, Universitas Indonesia, Jakarta, Indonesia

**Keywords:** COVID-19, Indonesia, Medical students, Readiness to practice, Willingness to volunteer

## Abstract

**Background:**

The question to involve or restrict medical students’ involvement in the coronavirus disease 2019 (COVID-19) pandemic response remains contentious. As their state of preparation and perceptions in volunteering during this pandemic have yet to be investigated, this study aims to evaluate Indonesian medical students’ willingness to volunteer and readiness to practice during the COVID-19 pandemic.

**Methods:**

A web-based survey was conducted among undergraduate medical students throughout Indonesia. Socio-demographic and social interaction information, in addition to willingness to volunteer and readiness to practice, were obtained using a self-reported questionnaire. The significance level was set at 5%.

**Results:**

Among 4870 participants, 2374 (48.7%) expressed their willingness to volunteer, while only 906 (18.6%) had adequate readiness to practice. Male students, students with prior volunteering experience in health or non-health sectors, and students from public universities or living in Central Indonesia (vs Java) had higher scores of willingness and readiness to volunteer. Students from Sumatra also had better preparedness (odds ratio [OR] 1.56, 95% confidence interval [CI] 1.15–2.12, *p* = 0.004), while the opposite occurred for students from Eastern Indonesia (OR 0.63, 95% CI: 0.44–0.89, *p* = 0.002)–when compared to students from Java. In addition, compared to students with high family income, students from lower-middle income families were less willing to volunteer (OR 0.76, 95% CI: 0.59–0.98, *p* = 0.034), though those with low family income had better readiness (OR 1.51, 95% CI: 1.10–2.08, *p* = 0.011). Shortage of medical personnel, sense of duty, and solicitation by stakeholders were the main reasons increasing the students’ willingness to volunteer; whereas contrarily fear for own’s health, absence of a cure, and fear of harming patients were the primary factors diminishing their willingness to volunteer.

**Conclusion:**

Our findings indicated that many Indonesian medical students are willing to volunteer, yet only few of them were ready to practice, indicating that further preparations are required to maximize their potentials and minimize their exposure to hazards. We suggest that their potentials as a firm support system during the pandemic should not be overlooked, and that the integration of relevant courses to the medical curricula are imperative to prepare for future public health emergencies.

## Introduction

The global coronavirus disease 2019 (COVID-19) pandemic has placed a significant burden on healthcare systems, resulting in millions of cases and deaths worldwide. Indonesia, the largest archipelago country in the world, has been the hardest-hit nation in Southeast Asia with several hundred thousands of cases and tens of thousands of deaths [1]. The current Indonesian health system is struggling to keep up with the perpetual COVID-19 case surges as recurrent shortage of medical supplies had been reported [2]. Furthermore, this was aggravated by the alarming rate of mental breakdown and death tolls among healthcare workers (HCWs) [3], thus raising concerns on the possibilities of medical staff shortages [2], in which the question remains whether to involve medical students as relief aids during this ongoing crisis or not [4].

The role of medical students during the COVID-19 pandemic remains contentious as their participation varies across countries. While some countries integrated their education into COVID-19 response systems, other countries specifically enforce policies to limit medical students’ involvement in medical practices [5]. In Indonesia, several student-led organizations have actively participated in the pandemic response by steering fundraising campaigns and providing continuing supports to HCWs. However, most of these initiatives were conducted in non-clinical settings, while their participation in medical care remains vague [6]. Although recently clerkship students have been assigned to teaching hospitals by implementing strict health protocols to facilitate their learning through direct patient exposures, their current involvement might not be sufficient reinforce HCWs given the ethical dilemma of their competence and patient care obligation [7]. Nonetheless, these enterprises signify the potentials of medical students as an integral part of the national healthcare system.

To the best of our knowledge, the willingness to volunteer and readiness to practice of medical students in Indonesia have yet to be investigated. In fact, the investigation of these aspects is saliently important in order to assess their preparedness to partake in pandemic responses and to shape the medical curricula to prepare for future public health emergencies [8, 9]. Therefore, this study aims to evaluate the willingness to volunteer and readiness to practice of undergraduate medical students in Indonesia during the COVID-19 pandemic.

## Methods

This study is part of a bigger project (“The willingness to volunteer and readiness of Indonesian medical students during the coronavirus disease 2019 pandemic”) conducted by the MEDICO-19 Research Group. The study protocol has been approved by the Health Research Ethics Committee, Faculty of Medicine Universitas Indonesia and Cipto Mangunkusumo National General Hospital (758/UN2.F1/ETIK/PPM.00.02/2020). All methods were performed in accordance with the relevant guidelines and regulations.

### Study design and participants

A cross-sectional survey was conducted among undergraduate medical students in Indonesia from 13 July to 11 October 2020. All data were collected online via a self-reported questionnaire using Google Forms (docs.google.com/forms). Participants were recruited with a snowball sampling method through social media and emails. Upon questionnaire distribution, participants were encouraged to forward the link to other relevant respondents.

The study population involved all undergraduate medical students in Indonesia with access to the internet. Prior to filling the questionnaire, respondents were provided with an online informed consent form, in which they had to answer a yes-no question to confirm their willingness to participate in this study. Respondents were informed that they had the rights to withdraw their consents at any time without any entailing consequences, and that all information would be kept anonymous and confidential throughout the study.

The minimum required sample size was calculated with an online sample size calculator (Raosoft, Inc., Seattle, WA) [10]. As we were unable to obtain the exact number of undergraduate medical students in Indonesia, the population size was estimated to be ~ 62,500 students. With a margin of error of 5% and a confidence level of 99%, a minimum of 657 participants were required in this study.

### Measurement tool and data management

Development of the questionnaire was performed through thorough literature search of similar studies [11–14], which was then modified to match the relevant context, validated, and presented to the respondents in Bahasa Indonesia. The initial survey was drafted in English and was translated to Bahasa Indonesia through forward and backward translation by GL, AMP, and IA – in order to ensure the language validity of the questionnaire. Validation of the questionnaire was first performed by two independent experts (AF and ISW) to ascertain the appropriateness and sufficiency of the contents. Subsequently, the questionnaire was pilot tested on 30 participants, in which their responses were obtained for items comprehension and validity and reliability testing [15].

The questionnaire consisted of four primary sections: socio-demographical variables, social interaction history, willingness to volunteer, and readiness to practice. Socio-demographic characteristics including age and sex, institution, level of education, household conditions, marital status, socioeconomic status, and history of chronic diseases were obtained in this study. Socioeconomic status was determined according to family income, where family income of ≤IDR 1,500,000, IDR 1,500,000-2,500,000, IDR 2,500,000-3,500,000, and ≥ IDR 3,500,000 were classified as low, lower-middle, upper-middle, and high-income class, respectively [16].

The second section aimed to investigate the respondents’ volunteering experience as well as COVID-19 contact and disease history. The third and fourth sections were presented in five-point Likert scales ranging from “strongly disagree” to “strongly agree”. The third section consisted of respondents’ willingness to volunteer (if healthy and able to), and possible reasons underlying their willingness and reluctance to volunteer. COVID-19 volunteering opportunity was defined as any voluntary activities, regardless of its sectors (i.e. health or non-health), aiming to alleviate the burden of the COVID-19 pandemic [17]. The final section aimed to assess the respondents’ readiness to volunteer [12] through 11 questions (maximum score: 55 points), where higher scores indicated better preparedness. Readiness to practice was determined from the respondents’ confidence in volunteering and state of preparation as COVID-19 volunteers. This section oriented the development of professional identity formation among undergraduate medical students during the COVID-19 pandemic, including their adaptations and coping strategies, perceived roles during the pandemic, and their extent of readiness to take part in the COVID-19 management, which encompasses prior experience in volunteering, knowledge and understanding on COVID-19, and COVID-19 courses enrolled [12, 18]. Items in the fourth section were tested for internal reliability using Cronbach’s alpha, which resulted in a coefficient of 0.767, indicating high internal reliability [19].

### Statistical analysis

Submitted responses were extracted from Google Forms to the MS Excel® for Office 365 MSO ver. 2002 (Microsoft Corporation, Redmond, WA, 2018) for cleaning and coding. Subsequently, the cleaned dataset was analysed using SPSS 24.0 (SPSS Inc., Chicago, IL) and visualized using R ver. 4.0.0 (R Foundation for Statistical Computing, Vienna, Austria) [20] with an additional *ggplot2* package [21]. Dichotomous data were presented in frequencies and proportions, while continuous data in mean ± standard deviation (SD) or median (interquartile range [IQR]), depending on data normality as tested with Kolmogorov-Smirnov tests.

Analysis of willingness to volunteer and readiness to practice was performed by dichotomizing the scores by cut-off points. According to the Bloom’s criteria [22], sum scores of ≥80% (≥44 points) indicated good readiness to practice. For willingness to volunteer, score of 1–3 denoted hesitance to volunteer, while score of 4–5 indicated likeliness to be willing to volunteer [23]. The association of independent variables with willingness to volunteer and readiness to practice was analysed using asymptotic Pearson’s chi-squared or Monte Carlo exact tests (for dichotomous variables) and Mann-Whitney U (for continuous variables). Covariates associated with each outcome at *p* ≤ 0.20 were included in the multivariate analysis. In addition, to explore the reasons influencing the participants’ willingness to volunteer, Goodman and Kruskal’s gamma statistic (G) was utilized. The significance level was set at 5% for all analyses.

## Results

### Characteristics of study population

A total of 4870 valid participants with a median age of 20 years (IQR: 19–21) were included for analyses. Most of the respondents were female (69.8%), and a majority resided in Java (86.9%). About 80.6% participants were still in their pre-clinical years, while the proportion of those from public and private universities was almost equal (Table 1). In terms of socioeconomic status, most respondents were living with their families during questionnaire completion with a median household size of four people (IQR: 3–5 people). In addition, most participants were not living with elderlies (76.3%), were not married (99.5%), and did not have any history of chronic illness (93.5%), whereas almost half of them were living with children. The majority of the participants were from high socioeconomic groups (78.5%).

**Table 1:** Characteristics of participants stratified by willingness to volunteer and readiness to practice^a^

Variable	N (%)	Willingness to volunteer; n(%)			Readiness to practice; n (%)		
		Unlikely; 2496 (51.3)	Likely; 2374 (48.7)	***P***-value	Poor; 3964 (81.4)	Good; 906 (18.6)	***P***-value
**Age (years)**	**20 (19–21)**	**20 (19–21)**	**20 (19–21)**	**0.076**^**b**^	**20 (19–21)**	**20 (19–21)**	**0.004**^**b**^
**Sex**							
**Male**	**1471 (30.2)**	**718 (48.8)**	**753 (51.2)**	**0.025**	**1134 (77.1)**	**337 (22.9)**	**< 0.001**
**Female**	**3399 (69.8)**	**1778 (52.3)**	**1621 (47.7)**		**2830 (83.3)**	**569 (16.7)**	
**Place of residence**	***N*** **= 4831**						
**Java**	**4199 (86.9)**	**2189 (52.1)**	**2010 (47.9)**	**< 0.001**	**3445 (82.0)**	**754 (18.0)**	**< 0.001**
**Sumatra**	**237 (4.9)**	**100 (42.2)**	**137 (57.8)**		**169 (71.3)**	**68 (28.7)**	
**Central Indonesia**^**c**^	**103 (2.1)**	**31 (30.1)**	**72 (69.9)**		**69 (67.0)**	**34 (33.0)**	
**Eastern Indonesia**^**d**^	**292 (6.0)**	**158 (54.1)**	**134 (45.9)**		**249 (85.3)**	**43 (14.7)**	
**Institution type**	**N = 4831**						
**Public**	**2525 (52.3)**	**1194 (47.3)**	**1331 (52.7)**	**< 0.001**	**1995 (79.0)**	**530 (21.0)**	**< 0.001**
**Private**	**2306 (47.7)**	**1284 (55.7)**	**1022 (44.3)**		**1937 (84.0)**	**369 (16.0)**	
**Academic level**							
**Pre-clinical**	**3925 (80.6)**	**2023 (51.5)**	**1902 (48.5)**	**0.411**	**3233 (82.4)**	**692 (17.6)**	**< 0.001**
**Clinical**	**945 (19.4)**	**473 (50.1)**	**472 (49.9)**		**731 (77.4)**	**214 (22.6)**	
**Living with**^**e**^							
**Family**	**4154 (85.3)**	**2146 (51.7)**	**2008 (48.3)**	**0.352**	**3401 (81.9)**	**753 (18.1)**	**0.084**
**Non-family**	**58 (1.2)**	**30 (51.7)**	**28 (48.3)**		**48 (82.8)**	**10 (17.2)**	
**Alone**	**658 (13.5)**	**320 (48.6)**	**338 (51.4)**		**515 (78.3)**	**143 (21.7)**	
**Number of housemate (people)**^**e**^	**4 (3–5)**	**4 (3–5)**	**4 (3–5)**	**0.679**^**b**^	**4 (3–5)**	**4 (3–5)**	**0.646**^**b**^
**Living with children**							
**Yes**	**2282 (46.9)**	**1153 (50.5)**	**1129 (49.5)**	**0.341**	**1879 (82.3)**	**403 (17.7)**	**0.112**
**No**	**2588 (53.1)**	**1343 (51.9)**	**1245 (48.1)**		**2085 (80.6)**	**503 (19.4)**	
**Living with elderly**							
**Yes**	**1155 (23.7)**	**603 (52.2)**	**552 (47.8)**	**0.457**	**936 (81.0)**	**219 (19.0)**	**0.721**
**No**	**3715 (76.3)**	**1893 (51.0)**	**1822 (49.0)**		**3028 (81.5)**	**687 (18.5)**	
**Marital status**							
**Married**	**21 (0.4)**	**13 (61.9)**	**8 (38.1)**	**0.233**^**f**^	**16 (76.2**	**5 (23.8)**	**0.716**^**f**^
**Not married**	**4847 (99.5)**	**2481 (51.2)**	**2366 (48.8)**		**3946 (81.4)**	**901 (18.6)**	
**Divorced**	**2 (0.0)**	**2 (100)**	**0 (0.0)**		**2 (100)**	**0 (0.0)**	
**Family income**							
**≤ IDR 1,500,000**	**241 (4.9)**	**122 (50.6)**	**119 (49.4)**	**0.096**	**178 (73.9)**	**63 (26.1)**	**0.016**
**IDR 1,500,000-2,500,000**	**273 (5.6)**	**153 (56.0)**	**120 (44.0)**		**227 (83.2)**	**46 (16.8)**	
**IDR 2,500,000-3,500,000**	**534 (11.0)**	**293 (54.9)**	**241 (45.1)**		**431 (80.7)**	**103 (19.3)**	
**≥ IDR 3,500,000**	**3822 (78.5)**	**1928 (50.4)**	**1894 (49.6)**		**3128 (81.8)**	**694 (18.2)**	
**History of chronic ilness**							
**Yes**	**316 (6.5)**	**165 (52.2)**	**151 (47.8)**	**0.723**	**243 (76.9)**	**73 (23.1)**	**0.034**
**No**	**4554 (93.5)**	**2331 (51.2)**	**2223 (48.8)**		**3721 (81.7)**	**833 (18.3)**	
**Volunteered in health sectors**							
**Yes**	**864 (17.7)**	**323 (37.4)**	**541 (62.6)**	**< 0.001**	**594 (68.8)**	**270 (31.3)**	**< 0.001**
**No**	**4006 (82.3)**	**2173 (54.2)**	**1833 (45.8)**		**3370 (84.1)**	**636 (15.9)**	
**Volunteered in non-health sectors**							
**Yes**	**3149 (64.7)**	**1454 (46.2)**	**1695 (53.8)**	**< 0.001**	**2460 (78.1)**	**689 (21.9)**	**< 0.001**
**No**	**1721 (35.3)**	**1042 (60.5)**	**679 (39.5)**		**1504 (87.4)**	**217 (12.6)**	
**Family members diagnosed with COVID-19**							
**Yes**	**387 (7.9)**	**182 (47.0)**	**205 (53.0)**	**0.057**	**302 (78.0)**	**85 (22.0)**	**0.200**
**No**	**3831 (78.7)**	**1958 (51.1)**	**1873 (48.9)**		**3132 (81.8)**	**699 (18.2)**	
**Don’t know**	**652 (13.4)**	**356 (54.6)**	**296 (45.4)**		**530 (81.3)**	**122 (18.7)**	
**Physical contacts with COVID-19 patients**							
**Yes**	**167 (3.4)**	**72 (43.1)**	**95 (56.9)**	**0.060**	**127 (76.0)**	**40 (24.0)**	**0.045**
**No**	**3618 (74.3)**	**1850 (51.1)**	**1768 (48.9)**		**2971 (82.1)**	**647 (17.9)**	
**Don’t know**	**1085 (22.3)**	**574 (52.9)**	**511 (47.1)**		**866 (79.8)**	**219 (20.2)**	
**Had been a COVID-19 patient**							
**Yes**^**g**^	**33 (0.7)**	**13 (39.4)**	**20 (60.6)**	**0.228**	**24 (72.7)**	**9 (27.3)**	**0.301**
**No**	**3990 (81.9)**	**2062 (51.7)**	**1928 (48.3)**		**3259 (81.7)**	**731 (18.3)**	
**Don’t know**	**847 (17.4)**	**421 (49.7)**	**426 (50.3)**		**681 (80.4)**	**166 (19.6)**	

^a^Unless otherwise stated, data are presented in n (%), mean ± standard deviation, or median (interquartile range), and analysed with chi-square tests; text in bold indicates statistical significance. ^b^Statistical significance derived from Mann-Whitney U test. ^c^Includes Sulawesi and Kalimantan. ^d^Includes Bali, Nusa Tenggara, Maluku, and Papua. ^e^Defined as the people living with the respondents at the time of questionnaire completion. ^f^Statistical significance derived from Monte Carlo exact method. ^g^Includes both confirmed and unconfirmed (suspected or probable) cases. *COVID-19* Coronavirus disease 2019, *IDR* Indonesian Rupiah

A large share of the participants had had experience as volunteers in non-health sectors (64.7%), while only 17.7% stated that they had volunteered in health sectors. Intriguingly, a remarkable number of participants stated that they did not know their own and family’s history of COVID-19 infection (17.4 and 13.4%, respectively), neither did they know about their history of direct physical contact with COVID-19 patients (22.3%). Only 3.4% participants reported that they had engaged in physical contacts with COVID-19 patients, and only 0.7% had been infected by the COVID-19. Further details on the participants’ characteristics can be seen on Table 1.

### Prevalence of and factors associated with willingness to volunteer and readiness to practice

Our findings indicated that nearly half of the participants were willing to volunteer during the COVID-19 pandemic (48.7%), including 626 participants (12.9%) who expressed their strong willingness. However, only 906 respondents (18.6%) yielded adequate readiness score. Willingness to volunteer varied by sex, location, institution type, and volunteering experience, whereas readiness to practice varied across age, sex, location, institution type, academic level, family income, history of chronic illness, volunteering experience, and history of physical contacts with COVID-19 patients (Table 1).

**Fig. 1:**
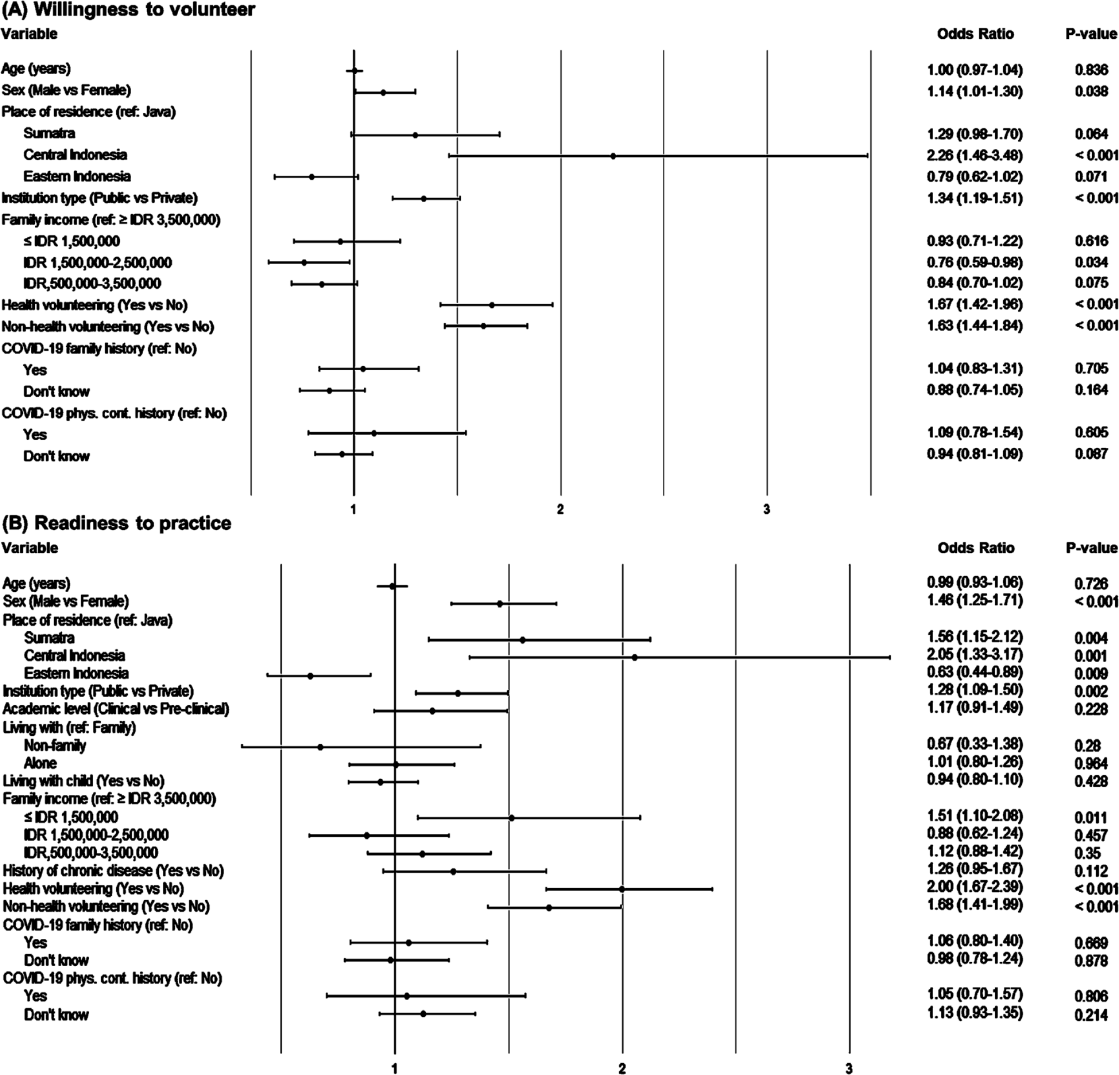
Results of multivariate logistic analysis on factors associated with Indonesian undergraduate medical students’ willingness to volunteer and readiness to practice. COVID-19, coronavirus disease 2019; COVID-19 phys. cont. history, history of physical contacts with COVID-19 patients; IDR, Indonesian Rupiah

After adjusting for confounders, we discovered that sex, location, institution type, family income, and volunteering experience were independent determinants of willingness to volunteer (Table 2). Male students and students living in Central Indonesia (i.e. Kalimantan and Sulawesi) were 1.14 (95% CI: 1.01–1.23, *p* = 0.038) and 2.26 (95% CI: 1.46–3.48, *p* < 0.001) times more likely to be willing to volunteer, while those from lower-middle families were 24.2% less likely to be willing to volunteer during the pandemic (OR 0.76 [95% CI: 0.59–0.98], *p* = 0.034)–when compared to those from high-income families. Additionally, students with prior volunteering experience in health or non-health sectors were more likely to be willing to volunteers (OR 1.67 [95% CI: 1.42–1.96] and OR 1.63 [95% CI: 1.44–1.84], respectively; both *p* < 0.001; Fig. 1a).

**Table 2:** Multivariate analysis on factors associated with willingness to volunteer and readiness to practice^a^

Variable	Willingness to volunteer			Readiness to practice		
	OR	95% CI	***P***-value	OR	95% CI	***P***-value
**Age (years)**	**1.00**	**[0.97, 1.04]**	**0.836**	**0.99**	**[0.93, 1.06]**	**0.726**
**Sex**						
**Male**	**1.14**	**[1.01, 1.30]**	**0.038**	**1.46**	**[1.25, 1.71]**	**< 0.001**
**Female**	**ref**			**ref**		
**Place of residence**						
**Java**	**ref**			**ref**		
**Sumatra**	**1.30**	**[0.99, 1.70]**	**0.064**	**1.56**	**[1.15, 2.12]**	**0.004**
**Central Indonesia**^**b**^	**2.26**	**[1.46, 3.48]**	**< 0.001**	**2.05**	**[1.33, 3.17]**	**0.001**
**Eastern Indonesia**^**c**^	**0.79**	**[0.62, 1.02]**	**0.071**	**0.63**	**[0.44, 0.89]**	**0.009**
**Institution type**						
**Public**	**1.34**	**[1.19, 1.51]**	**< 0.001**	**1.28**	**[1.09, 1.50]**	**0.002**
**Private**	**ref**			**ref**		
**Academic level**						
**Pre-clinical**				**ref**		
**Clinical**				**1.17**	**[0.91, 1.49]**	**0.228**
**Living with**^**d**^						
**Family**				**ref**		
**Non-family**				**0.67**	**[0.33, 1.38]**	**0.280**
**Alone**				**1.01**	**[0.80, 1.26]**	**0.964**
**Living with children**						
**Yes**				**0.94**	**[0.80, 1.10]**	**0.428**
**No**				**ref**		
**Family income**						
**≤ IDR 1,500,000**	**0.93**	**[0.71, 1.22]**	**0.616**	**1.51**	**[1.10, 2.08]**	**0.011**
**IDR 1,500,000-2,500,000**	**0.76**	**[0.59, 0.98]**	**0.034**	**0.88**	**[0.62, 1.24]**	**0.457**
**IDR 2,500,000-3,500,000**	**0.84**	**[0.70, 1.02]**	**0.075**	**1.12**	**[0.88, 1.42]**	**0.350**
**≥ IDR 3,500,000**	**ref**			**ref**		
**History of chronic disease**						
**Yes**				**1.26**	**[0.95, 1.67]**	**0.112**
**No**				**ref**		
**Volunteered in health sectors**						
**Yes**	**1.67**	**[1.42, 1.96]**	**< 0.001**	**2.00**	**[1.67, 2.39]**	**< 0.001**
**No**	**ref**			**ref**		
**Volunteered in non-health sectors**						
**Yes**	**1.63**	**[1.44, 1.84]**	**< 0.001**	**1.68**	**[1.41, 1.99]**	**< 0.001**
**No**	**ref**			**ref**		
**Family members diagnosed with COVID-19**						
**Yes**	**1.05**	**[0.83, 1.31]**	**0.705**	**1.06**	**[0.80, 1.40]**	**0.669**
**No**	**ref**			**ref**		
**Don’t know**	**0.88**	**[0.74, 1.05]**	**0.164**	**0.98**	**[0.78, 1.24]**	**0.878**
**Contacts with COVID-19 patients**						
**Yes**	**1.10**	**[0.78, 1.54]**	**0.605**	**1.05**	**[0.70, 1.57]**	**0.806**
**No**	**Ref**			**ref**		
**Don’t know**	**0.94**	**[0.81, 1.09]**	**0.411**	**1.13**	**[0.93, 1.35]**	**0.214**

^a^Text in bold indicates statistical significance. ^b^Includes Sulawesi and Kalimantan. ^c^Includes Bali, Nusa Tenggara, Maluku, and Papua. ^d^Defined as the people living with the respondents at the time of questionnaire completion. *CI* Confidence interval, *COVID-19* Coronavirus disease 2019, *IDR* Indonesian Rupiah, *OR* Odds ratio

Similar to willingness to volunteer, male students (OR 1.46 [95% CI: 1.25–1.71], *p* < 0.001), public university students (OR 1.28 [95% CI: 1.09–1.50], *p* = 0.002), and students living in Central Indonesia also had better preparedness (OR 2.05 [95% CI: 1.33–3.17], *p* = 0.001; Fig. 1b). Interestingly, students residing in Sumatra were also more likely to yield better readiness when compared to students living in Java (OR 1.56 [95% CI: 1.15–2.12], *p* = 0.004), while the opposite trend was observed for students living in Eastern Indonesia (OR 0.629 [95% CI: 0.44–0.89], *p* = 0.009). Students with experience as health or non-health volunteers also had higher readiness score (OR 2.00 [95% CI: 1.67–2.39] and OR 1.68 [95% CI: 1.41–1.99], both *p* < 0.001). Lastly, we also revealed that students with low family income was 51.2% more likely to be ready to practice during the COVID-19 pandemic (OR 1.51 [95% CI: 1.10–2.08], *p* = 0.011).

### Possible reasons underlying hesitancy to volunteer

To further elaborate our findings on the willingness to volunteer, we queried several common reasons that might potentially influence the decision of undergraduate medical students to take part in the COVID-19 response system. We discovered that shortage of medical personnel and self-obligation were the main drivers in determining the willingness to volunteer (G = 0.689 and G = 0.607, respectively; both *p* < 0.001). In addition, solicitation to volunteer by the government or stakeholders and sanctions for hesitance to volunteer also yielded positive correlation with willingness to volunteer (Table 3). Astoundingly, remuneration had no effect on medical students’ decision to volunteer (G = − 0.014, *p* = 0.462), though provision of monetary compensation when their services resulted in infection or death seemed to increase their willingness to volunteer (G = 0.062, *p* = 0.001 and G = 0.114, *p* < 0.001; respectively).

**Table 3:** Reasons associated with willingness and hesitance to volunteer

Reasons	Willingness to volunteer	
	Gamma	***P***-value
**Reasons for willing to volunteer**		
**Remuneration**	**-0.014**	**0.462**
**Compensated by money if infected by COVID-19**	**0.062**	**0.001**
**Compensated by money if death due to COVID-19 occurs**	**0.114**	**< 0.001**
**Shortage of medical personnel**	**0.689**	**< 0.001**
**Obligation as a medical student**	**0.607**	**< 0.001**
**Solicited by the government or medical institutions**	**0.648**	**< 0.001**
**Sanctioned if not willing to volunteer**	**0.211**	**< 0.001**
**Reasons for hesitance to volunteer**		
**Fear for own’s health**	**-0.221**	**< 0.001**
**Fear for family’s health**	**-0.048**	**0.021**
**Fear of harming patients**	**−0.117**	**< 0.001**
**Inadequate PPE**	**−0.059**	**0.002**
**Absence of a cure**	**−0.205**	**< 0.001**

*COVID-19* Coronavirus disease 2019, *PPE* Personal protective equipment

On the other hand, the most important reasons underlying the students’ reluctance to volunteer were fear for own’s health and the absence of a cure (G = − 0.221 and G = -0.205, respectively; both *p* < 0.001). Moreover, other reasons including fear of harming patients (G = -0.117, *p* < 0.001), followed by scarcity of personal protective equipment (PPE; G = -0.059, *p* = 0.002) and fear for family’s health (G = − 0.048, *p* = 0.021) also affected their willingness to volunteer.

## Discussion

As future healthcare professionals, medical students are regarded as those with closest capability to take part in battling the current pandemic [9]. Nevertheless, their potentials in fighting this pandemic, especially in Indonesia, have yet to be well-investigated. According to our findings, we discovered that a considerable number of medical students was willing to voluntarily support the care system during the pandemic. However, this was not accompanied by a proportional number of participants with adequate readiness to practice, indicating that further preparations are required to ensure that they are equipped with sufficient knowledge and aptitude [9]. This is in accordance to the findings of previous studies in Ireland [24] and Germany [5] which reported high rates of willingness to volunteer among medical students [5, 24] with relatively low rates of readiness to practice [24].

Further analysis revealed that place of residence and institution type played significant roles in determining willingness to volunteer and readiness to practice. These effects may potentially be elucidated by the fact that state-university students and students residing in Central Indonesia and Sumatra had higher rate of prior health volunteering experience (public vs private: 21.1% vs 13.8%; Central Indonesia vs Java: 23.3% vs 16.8%; Sumatra vs Java: 28.3% vs 16.8%). However, the interaction between these variables are complex, and future investigations should aim to elaborate possible reasons underlying these trends. Moreover, we also observed that male students had higher willingness and readiness to volunteer. Although the rationale of this interplay has yet to be discovered, our findings were consistent with previous studies [25, 26], thus further consolidating the evidence. Our findings also indicated that students with lower family income had lower rates of willingness to volunteer, which was concordant with the previous findings by Niebuur et al. [27]. Despite this, these students yielded higher score in readiness to practice, which may result from their prior experience on the privilege gap in medicine [28].

In addition, volunteering experience, either in health or non-health sectors, was also one of the strongest determinants influencing the willingness to volunteer and readiness to practice of Indonesian medical students. One possible explanation is that volunteering activities may increase their values (selfless virtues), understanding (knowledge and experience), enhancement (self-satisfaction), career (career-related experience), social (fortification of social relationship), and protection (alleviation of negative feelings or personal problems) [29], which may create a positive feedback loop resulting in increased willingness and readiness. Furthermore, these students are also more likely to be affiliated with volunteering organizations, thus further acclimatizing their environment to volunteerism [13]. These findings imply that medical institution and relevant stakeholders should encourage students to take part in volunteering activities to enrich them with invaluable knowledge and experience, which in turn may also be beneficial for the students’ values and career [29].

We observed that moral values linked to medical profession were the primary reason underlying the willingness of medical students in Indonesia to volunteer during the pandemic. Their altruism was expressed by their strong willingness to volunteer if medical staff shortage occurs or if they were requested to volunteer. Furthermore, monetary incentives were not associated with increased willingness – further signifying their selfless traits. On the contrary, most students expressed concerns for potential harms to their own and family’s health; in addition to paucity of PPE, which also reduced their willingness to volunteer, indicating that a systematic planning of allocation of volunteers and assurance of adequate PPE are required to prevent them from being exposed to unnecessary risks [9]. In addition, the fact that fear of causing harm to patients still influenced their decisions further emphasized that proper trainings are urgently needed. Clinical confidence as one of prerequisites to fully participate in clinical activities is reported to be influenced by adequate training programs [30], which is also vital to tackle global health emergencies [31]. The altruistic attitude of medical students, when equipped by adequate training and protection, may foretell the potential force of medical students as a firm support to the national health system.

Altogether, our findings indicated that the potential influence of Indonesian medical students in mitigating the COVID-19 pandemic should not be overlooked. However, further preparations are essential to ensure that they can volunteer effectively without exposing them to unnecessary harms. These preparations may be achieved through distance learning and practical activities which enable students to engage with similar real-world circumstances [32]. The feasibility of such programs have been proven by Christensen et al. [33], which stated that online trainings on equipping and disposing PPE resulted in comparable performances to offline instructor-led trainings. In Indonesia, a medical institution has implemented a massive open online course (MOOC) to equip medical students with essential biomedical, clinical, and epidemiologic knowledge on COVID-19. Furthermore, the course also provided basic skills trainings including infection prevention and control, mass education, and risk communication through video lessons and webinar sessions [34]. The use of MOOCs during the current pandemic have been shown to be beneficial in strengthening healthcare systems [35]. In addition, these efforts may not only equip them with adequate knowledge and skills, but also potentially inspiring them to further contribute to the healthcare system, thus ultimately benefitting both the students and the communities [13]. Nonetheless, the implementation of such preparations does not necessary imply that medical students may become a reserve for HCWs during an infectious outbreak. In fact, several studies had consistently reported that medical students were concerned about approaching patients infected with unknown diseases. In such cases, it may be plausible to focus the allocation of these volunteering students to activities relating to early levels of prevention with minimum risk of exposure, including health education and promotion, contact tracing and triages, as well as peer supports for isolating patients and HCWs [5, 36, 37]. Contrarily, volunteering opportunity in clinical settings should be strictly limited to clinical-year students, provided that they are well-prepared and protected [32].

Although it is true that the current pandemic may hinder the implementation of conventional medical education, this pandemic also carries an enormous learning opportunity, especially for final-year medical students, as proven by several institutions which had managed to successfully integrate their students to directly partake in pandemic responses [5, 32]. These students may take part in contact tracing, triage, diagnostic swab-testing, and even medical care support, which in turn may provide invaluable experience in collaborative patient care and benefit their career as future physicians [5, 37]. Nonetheless, it should be noted that their trainings and allocated role as volunteers should be based on their core competencies, and their integration to COVID-19 pandemic response system should be prioritized to clinical-year students as they are perceived to yield the most adequate knowledge and experience in clinical practice [7, 32]. Furthermore, their participation should always be voluntary and should satisfy their personal requirements [38].

The current pandemic further emphasizes the need of global health and disaster preparedness among Indonesian medical students. Hence, it is imperative for medical institutions to integrate student-centred and systematic courses to the undergraduate curricula – both in pre-clinical and clinical stages, to ensure that prospective health professionals are adequately prepared for future public health emergencies [8, 9]. These courses should strive to develop skills on multidisciplinary preparedness, health information management, emergency decision making, and leadership during crisis, in addition to basic knowledge and competencies related to global health emergency preparedness, such as basic life support, vaccination, and triage [9, 31, 39]. Furthermore, emphasis should be placed on integrating global health and disaster preparedness courses with the current available programs while still explicitly maintaining the curriculum process to achieve relevant competencies [40]. In the context of Indonesia, with a large number of highly-diverse medical schools, recognition of global health emergency-related competencies in the national competency standards and other learning resources are essential to tackle the need of a more sustainable medical curricula [40, 41].

The strength of our study lies in the relatively large sample size, which enabled us exhaustively explore factors associated with willingness to volunteer and readiness to practice. However, it should be noted that most of our participants resided in Java; which, although it reflected the current proportion of medical schools in Indonesia (45 out of 89 schools [42]), it potentially limited the representability of our findings. Furthermore, this study was limited due to its cross-sectional design, suggesting the inability to explain causations between variables. There are also possibilities that other factors (e.g., ethnicity and religion), which were unexplored in this study, might potentially confound our findings, thus future studies should also aim to explore the interplay of these factors. To the extent of our knowledge, this is the first study investigating the willingness and readiness of Indonesian medical students to participate in the COVID-19 response system, thus providing indispensable insights to policymakers to maximize the potentials of these students as an unyielding support to the national health system and to better prepare for future public health emergencies.

## Conclusion

Many undergraduate medical students in Indonesia were willing to volunteer during the COVID-19 pandemic. However, further preparations are required as only few students yielded satisfactory readiness score. We discovered that sex, prior volunteering experience, type of university, place of residence, and socioeconomic status were independent factors associated with willingness to volunteer and readiness to practice. We also revealed that shortage of medical staffs and moral obligations were the primary reasons underlying their willingness to volunteer, whilst fear for personal health and absence of a cure were the strongest motives negatively impacting their willingness to volunteer.

Our findings suggested that proper trainings and protections are urgently required to ensure that these students can volunteer effectively in this pandemic, and that policymakers should not overlook the potential force of medical students as a support to the health system. Medical institutions should also seek to integrate global health and disaster medicine to the medical curricula, thus contributing to the mitigation of future public health emergencies.

## Data Availability

The datasets generated and/or analysed during the current study are not publicly available due to the government’s data sharing policy which we abide by, since our project was funded by the government, but are available from the corresponding author on reasonable request.

## Abbreviations

COVID-19: Coronavirus disease 2019
CI: Confidence interval
HCW: Healthcare worker
IQR: Interquartile range
OR: Odds ratio
PPE: Personal protective equipment
SD: Standard deviation
